# Promoting children’s science, technology, engineering, and mathematics learning at home through tinkering and storytelling

**DOI:** 10.3389/fpsyg.2023.1146063

**Published:** 2023-05-03

**Authors:** Maria Marcus, Graciela Solis, Shelby Sellars, Catherine A. Haden

**Affiliations:** ^1^Department of Psychology, Roosevelt University, Chicago, IL, United States; ^2^Department of Psychology, Loyola University Chicago, Chicago, IL, United States

**Keywords:** storytelling, STEM learning, parent-child interactions, memory, informal learning, museums

## Abstract

This study examined whether connecting storytelling and tinkering can advance early STEM (science, technology, engineering, and mathematics) learning opportunities for children. A total of 62 families with 4- to 10-year-old (*M* = 8.03) children were observed via Zoom. They watched a video invitation to tinker at home prepared by museum educators prior to tinkering. Then, half of the families were prompted to think up a story before tinkering (story-based tinkering group), whereas the other half were simply asked to begin tinkering (no-story group). Once they had finished tinkering, researchers elicited children’s reflections about their tinkering experience. A subset of the families (*n* = 45) also reminisced about their tinkering experience several weeks later. The story instructions provided before tinkering engendered children’s storytelling during tinkering and when reflecting on the experience. Children in the story-based tinkering group also talked the most about STEM both during tinkering, and subsequently when reminiscing with their parents about their tinkering experience.

## Introduction

In this study, our goal is to understand how storytelling can be integrated into informal science, technology, engineering, and mathematics (STEM) learning experiences for children at home. Although most of the research and educational practices involving stories concern developing language and literacy skills, there is growing interest in and evidence for stories fostering children’s STEM learning (see Haden et al., 2023, for review). This is important because it contributes to a broader effort in the United States to design and implement educational opportunities that can build competencies in STEM and support future STEM-related pursuits (National Research Council [NRC], 2012; NGSS Lead States, 2013). In addition to education in schools, informal learning experiences in homes, museums, and libraries can promote the development of skills and competencies that are important in STEM fields (National Academy of Engineering [NAE] and National Research Council [NRC], 2009; National Research Council [NRC], 2009, National Research Council [NRC], 2012; Sobel and Jipson, 2016; NAS, 2018). In terms of specific STEM-related activities, one that has been underscored by educators and policymakers is *tinkering,* a form of playful, hands-on problem solving involving everyday materials (Honey and Kanter, 2013; Bevan, 2017). Some argued that tinkering is a nearly ideal target for research and educational practices that aim to encourage STEM because of its “low floors” to get started, “high ceilings” that do not limit complexity of projects, and “wide walls” that can engage learners from many different backgrounds and interests (Resnick, 2016; Vossoughi et al., 2016 Acosta, 2022). However, to realize the potential for tinkering to foster children’s STEM learning, we must expand the range of educational practices that engage children in STEM-rich tinkering (Pagano et al., 2020). This is a primary aim of our work in which we ask the research question of whether prompting parent–child oral storytelling while participating in tinkering at home can advance informal STEM learning opportunities for children.

### Stories for promoting STEM learning

Children begin telling stories almost as soon as they begin talking, and stories in books, movies, videogames, and television shows, as well as oral stories in conversations with others are ubiquitous in the lives of children. A great deal of theory and research recommends stories for successful learning generally (e.g., Nelson, 1989; Bruner, 1996; Brown et al., 2014), as well as for science learning specifically (Avraamidou and Osborne, 2009; Frykman, 2009; Klassen, 2010; Dahlstrom, 2014; Browning and Hohenstein, 2015; Wilson-Lopez and Gregory, 2015). There is also growing emphasis on stories beyond books, including oral storytelling (Haden et al., 2023). This is in keeping with Bruner’s (1991) theorizing that oral stories are a natural way of conveying knowledge that perhaps is more engaging for children and adults than STEM-related texts. Importantly, broadening the focus on stories to include oral storytelling may harness cultural resources for supporting children’s STEM learning at home (Haden et al., 2023). For example, for families from Latin American heritage and other cultural communities with firmly rooted oral traditions, oral storytelling may be a more common everyday practice for conveying knowledge to young children than book reading (Sánchez, 2009; Melzi et al., 2019).

There are several reasons why stories can support rich opportunities for children’s STEM learning. For one, stories can convey science information that may not be available through direct hands-on experiences with objects, and foster children’s engagement with abstract and challenging STEM-related ideas (National Research Council [NRC], 2009; Kelemen et al., 2014; Browning and Hohenstein, 2015; Evans et al., 2016; Cho and Plummer, 2018). Additionally, stories follow a narrative structure that can add coherence to experiences and enhance understanding of causal relations (Bruner, 1991; Trabasso and Stein, 1997; Reese et al., 2011). In turn, more coherent representations of STEM information and experiences can support retention and transfer of STEM learning (Klassen, 2010; Dahlstrom, 2014). Stories can also ground hands-on STEM activities and abstract STEM-related concepts in meaningful, interesting, and accessible scenarios, and help children realize the utility and relevance of mathematical, scientific, and engineering concepts and problems in their everyday lives. Furthermore, drawing on sociocultural theories that emphasize social communicative exchanges between children and caregivers (e.g., Vygotsky, 1978; Rogoff et al., 2018), stories can promote elaborated conversations involving cognitively challenging language, scaffolding children’s engagement with STEM-related principles and practices (Solis and Callanan, 2018; Plummer and Cho, 2020; Shirefley et al., 2020). Whereas children can learn a lot through direct experience interacting with objects (Piaget, 1970; Vygotsky, 1978), the kinds of conversation that stories can engender may provide critical supports for learning (Jant et al., 2014). In sum, stories can strengthen STEM learning by making what gets into memory more concrete, coherent, and comprehensible, thereby offering powerful mechanisms for children’s STEM learning.

Evidence that stories can support STEM learning comes from work in schools and informal educational settings. For example, there are a number of early childhood curriculum and resources for teachers in schools that use stories to contextualize hands-on activities about mathematics, science, and engineering (e.g., Brophy et al., 2008; Casey et al., 2008; van den Heuvel-Panhuizen et al., 2009; Elia et al., 2010; Aguirre-Muñoz and Pantoya, 2016; Cunningham, 2018; English and Moore, 2018; Giamellaro and O’Connell, 2018; Stanford et al., 2021). For example, in the *Engineering is Elementary (EiE)* curriculum^1^ (Cunningham and Lachapelle, 2014), a unit on bridge building is introduced with a story about a boy named Javier who lives in Texas and explores the field of civil engineering so as to build a stronger bridge to his backyard fort. *EiE* reports pre- to post-test gains in understanding of the engineering design process, and benefits for students’ confidence and attitudes about future STEM-related education and careers choices (Cunningham, 2018). As another example, Casey et al. (2008) designed a series of block building activities that for one group of kindergarteners were paired with oral stories told by a teacher from a book. Children in the building + story condition, for instance, heard that Sneeze the dragon wanted a 2-blocks high wall around the castle grounds to help keep animals from jumping over. Children in the building only condition were invited to build an enclosure with the same constraints without the story context; those in the control condition participated in unstructured block building. Compared with children in build-only and control groups, those in the building + story condition showed the greatest pre- to post-test improvements in spatial skills that are positively associated with STEM abilities.

Our focus on stories and tinkering at home is further encouraged by work in informal educational settings (e.g., Luke et al., 2010; Murmann and Avraamidou, 2016; Pattison et al., 2022). Plummer and Cho (2020) designed story-driven science programs for preschoolers. For example, after reading *Moonbear’s Shadow*, a museum educator prompted children to investigate the relations between a light source (flashlight), object (plastic bear toy), and shadows, which led the children to co-construct evidence-based explanations. In Letourneau et al. (2022), 7- to 14-year-old girls were observed during museum-based engineering design activities that used elements of stories (characters, settings, problems) to prompt consideration of who and what their designs were for. The stories supported engagement in multiple engineering design practices, expressions of empathy for the characters, and the making of connections between the stories and the girls’ personal experiences. Tzou et al. (2019) invited Indigenous families to animate family oral stories using robotics and computer coding during library-based workshops. As families enacted their stories with the roboticized dioramas they created, the stories not only framed material exploration and design, but also motivated goals and fixes for story-related problems. Further, Solis et al. (2019) found that when library- and museum-based programs for families were led by engineering experts who told oral engineering stories to frame hands-on activities, families talked more about engineering when engaging in the activities. In turn, the children also reported more engineering information in their reflections about the activities immediately afterward.

Our consideration of stories that parents and children tell is based in prior work suggesting that if stories can engender STEM-rich conversations, STEM learning can result. The frequency of specific STEM-related language inputs, such as spatial and relational language and mathematical vocabulary, can predict children’s skills in STEM domains (Gunderson and Levine, 2011; Pruden et al., 2011; Hassinger-Das et al., 2015; Casasola et al., 2020). Likewise, work on family conversations in museums, libraries, and at home suggests that the content of parent–child conversational interactions can support children’s STEM learning (Crowley et al., 2001; Geerdts et al., 2015; Callanan et al., 2017; Eberbach and Crowley, 2017; Solis et al., 2019; Booth et al., 2020). For example, Willard et al. (2019) found that the more parents and children engaged in explanatory talk in a STEM-related museum exhibit the more children talked about causal mechanisms and engaged in STEM-related practices in the exhibit. Also, parent–child STEM talk during science and engineering activities in museum exhibits has been linked to children’s recall of STEM-related information immediately after exhibit experiences, and in conversations and activities at home days and weeks later (Benjamin et al., 2010; Leichtman et al., 2017; Marcus et al., 2017; Pagano et al., 2020; Acosta et al., 2021; Marcus et al., 2021; Sobel et al., 2022).

### The current study

In this study, we aimed to engage parents and children in storytelling during a tinkering activity that they participated in at home. Tinkering often involves everyday, familiar, and recyclable materials (e.g., cardboard, paper, glue, and tape)—things families have around their homes. Tinkering is also frequently social, involving multiple family members (Vossoughi and Bevan, 2014). Early STEM learning opportunities can be greatly enhanced when tinkering centers on participants’ own ideas and objectives, as opposed to other sorts of building activities where there is a set or prescribed outcome (e.g., building a house with pieces and directions from a kit; Bevan, 2017). Moreover, tinkering can connect with STEM-related principles and practices in a range of ways (Vossoughi and Bevan, 2014; Pagano et al., 2020; Acosta et al., 2021). For example, tinkering creates opportunities for families to engage in the engineering design process, including making something to address a problem or need, and iterating the design after testing it for success (Cunningham and Lachapelle, 2014: Vossoughi et al., 2016). Math and science engagement is evident during tinkering as well, such as when children talk about how high some part of the structure needs to be, explain the relations between the parts and the whole of a structure, and discuss their thinking about what might work or not work in making and iterating their creation (Diefes-Dux, 2015).

For this study, as part of a research-practice partnership between university researchers and informal STEM learning practitioners at Chicago Children’s Museum, educators created a videorecorded invitation for families to tinker at home. This was at the start of the COVID-19 pandemic when the museum temporarily closed to visitors. In the video, an educator invited families to tinker to make a playground ride for a toy friend using materials they had around their home, and encouraged engagement in storytelling and the engineering design process. In addition to the dissemination of the video invitation on the museum’s website and social media platforms, our team began recruiting research participants. With half of the families in the research sample, we further encouraged telling stories during tinkering (i.e., story-based tinkering) by providing some time to think up their story before tinkering. During tinkering, we measured whether and to what extent families were telling stories by measuring the frequency of story talk, as well as the frequency of STEM-related talk during tinkering.

Immediately after tinkering we invited children’s reflections about the tinkering experience. Reflection is both a crucial part of the learning process and a means for revealing learning outcomes (e.g., Marcus et al., 2021). Reflection is also foundational in modern STEM education (e.g., National Research Council [NRC], 2009; NGSS Lead States, 2013). Opportunities to reflect on hands-on activities shortly after they have taken place can support the process of consolidation, whereby ephemeral patterns of experience are strengthened and transformed into lasting memories (Pagano et al., 2019). It also seemed possible that children who engaged in story-based tinkering would engage in more story and STEM talk about their tinkering experience immediately afterward, potentially drawing on the story they had told, which might help organize their engagement in engineering design and other STEM-related practices and support their reports. Furthermore, we engaged children in reminiscing with their parents several weeks after the tinkering experience, following up with them again via Zoom. These reminiscing conversations offered a vantage point from which to assess what STEM-related information had been retained post-tinkering and whether those in the story-based tinkering group were better able to recall this information compared to the no-story group.

We tested several sets of hypotheses. First, we hypothesized that children and parents who were prompted to tell a story during tinkering would mention more story components and talk more about STEM during tinkering than those in the no-story condition. Secondly, we predicted that when compared to those in the no-story during tinkering condition, children in the story-based tinkering condition would talk more about story and STEM in their immediate reports after tinkering. Lastly, we hypothesized that children in the story-based tinkering condition would talk the most about STEM when reminiscing with their parents weeks after tinkering. Essentially, we expected that prompting storytelling during tinkering at home would support children’s understanding and remembering of STEM information and that this would be evident both in their immediate reflections and later post-tinkering reminiscing.

## Methods

### Participants

Sixty-two families with 4- to 10-year-old children (*M*_age_ = 8.03, SD = 1.72; 30 girls) participated in this study. We elected to focus on this age group because research shows that the preschool and early elementary years can be a crucial period for advancing STEM learning and interest (National Research Council [NRC], 2012). Also, this is the age group that our partners see in the museum’s Tinkering Lab exhibit, and the ages for whom they designed the online programming during the pandemic. Families were recruited with the help of Chicago Children’s Museum’s outreach efforts, by recontacting families previously observed at the museum in different studies, and through word of mouth. Based on parent report, 54.8% of the children were White, 14.5% Black, 8.1% Asian, 4.8% Latinx, and 16.1% mixed race/ethnicity (race/ethnicity information was missing for 1 child). Of the parents, 87% held a college degree or higher. Of the 57 families who reported family income, 14.5% earned $200,000 or more, 12.9% earned $150,000-199,999, 30.6% earned $100,000-149,999, 22.6% earned $75,000–$99,999, 9.7% earned $50,000-74,999, and 1.6% earned $25,000-49,999. Of the families, 56.5% participated in our previous studies.

### Procedure

The study was approved under Loyola University Chicago IRB protocol #2992, *Tinkering with Digital Storytelling.* A researcher met with each family individually via Zoom for two sessions which were video- and audiorecorded. The first session involved observations of parents and children tinkering at home followed by a researcher eliciting the children’s reflections. The second session was to record parent–child reminiscing conversations.

#### Observations of tinkering at home

Prior to the first Zoom meeting, parents gave consent and were provided with a list of suggested materials to collect in advance of our tinkering observation session. These materials included paper, cardboard, tape, string, and glue. At the outset of the first session, we spent a few moments with each family ensuring that the camera angle was such that we could observe the parents and children in the workspace they had selected in their homes to engage in the tinkering activity.

All parents and children watched via Zoom a 5-min video introduction to the tinkering activity. The video was created by educators at Chicago Children’s Museum and introduced the tinkering at home activity: to create a playground ride for a small toy friend.^2^ In the video, a museum educator introduced several steps to complete the tinkering activity, including choosing a small toy, planning, making the ride, and sharing a story about the toy and the ride. The video also described the engineering process of making, testing, and fixing the creation, and illustrated these practices with an example of a swing the educator made for her character “Crunch” (a cork with eyes drawn on it) from cardboard, rubber bands, sticks, string, and tape.

After participants viewed the video, the researcher explained to all families that they had 30 min to complete the tinkering activity. During tinkering, the researcher turned off their camera and microphone to avoid drawing attention to the videorecording. When the 30 min were up, all families were given the option of taking up to 5 more minutes to finish up.

Families were randomly assigned to either the story-based tinkering condition (*n* = 29) or the no-story tinkering condition (*n* = 33). Immediately after the video invitation to tinker concluded, the families in the no-story condition were invited to start tinkering. Those in the story-based tinkering condition were asked by the researcher to spend a few minutes thinking up their story about their toy friend and their playground ride, what the toy would ride on, and how they could make it fun and safe. Each of these elements had been mentioned in the video; the story-based tinkering condition was aimed at emphasizing the storytelling component of the activity, and to give families in this group time to develop their story before beginning tinkering. Once families in this condition said they were ready, they began the tinkering activity.

#### Children’s reflections immediately after tinkering

Immediately after tinkering, researchers invited all children to show off their creations. The researcher then elicited all children’s reflections about their tinkering experience through a series of questions: (1) Tell me all about what happened with your character today? Tell me all about what they were thinking and doing! (2) What did you make for your character? (3) How did you make it for them? (4) Did you test your project to see if it worked? (5) How did it turn out? (6) Did you have to change or fix anything? (7) What did you learn? Researchers followed up with “Tell me more” and other encouragement as the children provided their reports in response to the prompts. After the children’s immediate reflections, the researcher asked the parents to report demographic information (including parent education, family income, and parents’ occupation) and about children’s prior experiences.

#### Parent–child reminiscing

A researcher who had not observed the families for the first session conducted the second session via Zoom. Our protocol was for the second session to occur approximately 2 weeks after the initial tinkering session. The researcher invited the parents and children to talk about the tinkering activity from the very beginning to the very end the way they would normally talk about past experiences together.

### Coding

All coding was conducted based on video recordings and transcripts. For each coding system, two coders independently coded 20% of the data, scoring the parents’ and children’s talk separately, to establish interrater reliability.

#### Parents’ and children’s story talk

Development of the story coding scheme was informed by the work of Hickmann (2003) that emphasized the importance of maintaining and reintroducing characters as a crucial narrative skill that allows the children to chart the progress of characters and elaborate their roles as the plot progresses. We coded for the frequency of story talk, including questions or comments about *characters—*naming the toy friend or object representing the character the creation was being built for, describing personality characteristic (e.g., “She likes to ride fast.”), desires (e.g., “He wants it to be yellow.”), or talking for the toy; *settings*—naming locations or physical surroundings (e.g., “It’s in a park.” or “It’s on a beach.”), or mentioning imagined places (e.g., “It’s in a magical forest.”); *actions*—within the story such as descriptions of physical movements (e.g., “He climbed up the ladder.”) or explanations of the plot (e.g., “He’s waiting for his friend to come over.”); and *conflicts/problems*—about obstacles or challenges that the toy is facing while on the playground ride, or problems that the toy must overcome (e.g., “She is too small to reach the button.” or “Her hat keeps falling off.”). Interrater reliability using Cohen’s Kappa was 0.91 for parents’ talk and 0.88 for children’s talk during tinkering, and 0.92 for the children’s story talk in the reflections immediately after tinkering.

#### Parents’ and children’s STEM talk

Using a system adapted from prior work (Haden et al., 2014), we coded for the frequency of parents’ and children’s STEM talk, including questions or statements pertaining to *project naming—*what they were planning to build (e.g., “What do you want to make?; “I want to make a slide.”), *planning—*suggestions or ideas about what to use next, *modifying design*—about making adjustments and improvements to the design while constructing, such as discussing the stability, strength, or adding new elements (e.g., “You have to have a strong foundation around everything!” or “Let’s put this in the middle to make it more stable.”), *testing*—about trying out their design (e.g., “Do you want to try it on the slide?”; “I want to see if it moves.”), *iterating/improving—*about how to fix something that wasn’t working (e.g., “How can we fix this?” “I need to fix this seat.”), and *mathematics*—such as length, size, weight, height, measurement, distance, geometric shapes, and numbers (e.g., “We need 2 pieces of that strong string.”). Cohen’s Kappa averaged 0.86 for parents’ talk and 0.91 for children’s talk during tinkering, 0.88 for the children’s reports immediately after tinkering, and 0.82 and 0.81 for the parents and children, respectively when reminiscing.

## Results

### Preliminary analyses

Preliminary analyses examined whether children in the two conditions (story-based tinkering, no-story) were equivalent in terms of child age, gender, and prior experiences, as well as parents’ education and whether they had a STEM-related job. As would be expected based on our random assignment of participants to groups, there were no age differences between the children in the story-based tinkering (*M* = 7.86, SD = 1.71) and the no-story conditions (*M* = 8.18, SD = 1.74), *F*(1, 61) = 0.53, *p* = 0.469, *η^2^* = 0.01, nor were there any gender differences, *χ^2^*(1, *N* = 62) = 0.000, *p* = 0.99, Cramer’s V = 0.002. Likewise, children’s prior experiences as assessed at the end of the tinkering session via parent report did not differ across groups (see Supplementary Table S1 in the Supplementary material), all *Fs* ≤ 3.14, *p*s ≥ 0.082, *η^2^*s ≤ 0.05. We found no differences between conditions for parent education, *F*(1, 59) = 0.04, *p* = 0.852, *η^2^* = 0.00, and family income, *χ^2^*(5, *N* = 57) = 4.70, *p* = 0.453, Cramer’s V = 0.287, *p* = 0.453. Parents’ occupations were categorized as STEM (35.5%) or non-STEM (62.9%) according to the Occupational Information Network^3^ and there were no differences between the two conditions with respect to parents’ occupation, *χ^2^*(1, *N* = 61) = 0.68, *p* = 0.411, Cramer’s V = 0.11, *p* = 0.411. Therefore, these preliminary analyses indicated it was not necessary to control for any of these demographic and prior experiences variables in our main analyses.

We also examined whether families were engaging longer in the tinkering or the reminiscing conversations as a function of condition and found no differences. Specifically, families spent an average of 26 min tinkering. Families in the story-based tinkering group (*M* = 27.06, SD = 3.36) and the no-story tinkering group (*M* = 26.13, SD = 4.58) were not different in time spent tinkering, *F*(1, 61) = 0.63, *p* = 0.430, *η^2^* = 0.01. Further, families spent on average 6 min reminiscing about their tinkering experiences. Families in the story-based tinkering group (*M* = 6.18, SD = 4.14) and no-story tinkering group (*M* = 5.59, SD = 2.41), were not different in time spent reminiscing, *F*(1, 49) = 0.11, *p* = 0.745, *η^2^* = 0.00. Given these results, we did not control for time spent in our main analyses.

### Tinkering activity

We hypothesized that children and parents in the story-based tinkering group would mention more story components and talk more about STEM during tinkering than those in the no-story condition. To test our hypotheses, we conducted a series of one-way ANOVAs. As shown in Table 1, children in the story-based tinkering condition mentioned more story components than those in the no-story condition, *F*(1, 61) = 10.73, *p* = 0.002, *η^2^* = 0.15. Children in the story-based tinkering condition also talked more about STEM while tinkering than those in the no-story condition, *F*(1, 61) = 12.29, *p* = 0.001, *η^2^* = 17. However, in contrast to our hypotheses, parents in the two conditions did not differ in their talk about story components, *F*(1, 61) = 1.81, *p* = 0.183, *η^2^* = 0.03, or STEM, *F*(1, 61) = 0.41, *p* = 0.523, *η^2^* = 0.01. When we further examined correlations between parents’ and children’s talk, we found that their story talk was correlated, *r* = 0.56, *p* < 0.001, whereas parents’ and children’s STEM talk during tinkering was not, *r* = 0.01, *p* = 0.94.

**Table 1:** Comparison between families in the story-based and no-story tinkering conditions.

	Tinkering condition						
	Story-based		No-Story				
	*M*	SD	*M*	SD	*F*	*p*	*η* ^2^
Story talk during tinkering							
Parents’ story talk	6.07	3.25	5.06	2.65	1.81	0.183	0.03
Children’s story talk	8.48	4.30	5.00	4.07	10.73	0.002	0.15
STEM talk during tinkering							
Parents’ STEM talk	12.83	3.70	13.39	3.23	0.41	0.523	0.01
Children’s STEM talk	7.28	1.96	5.45	2.11	12.29	0.001	0.17
Children’s talk immediately after tinkering							
Children’s story talk	11.21	2.08	8.45	2.56	21.20	0.000	0.26
Children’s STEM talk	10.24	3.75	10.64	2.56	0.24	0.626	0.00
STEM talk during reminiscing							
Parents’ STEM talk	9.05	4.22	7.13	2.88	2.95	0.093	0.07
Children’s STEM talk	6.38	2.56	3.08	1.59	26.65	0.000	0.39

### Immediate reports

We hypothesized that in comparison to children in the no-story condition, those in the story-based tinkering condition would report more story components and STEM information to a researcher immediately after tinkering. As shown in the middle of Table 1, children in the story-based tinkering condition did mention more story components than those in the no-story group, *F*(1, 61) = 21.20, *p* < 0.001, *η^2^* = 0.26. However, there were no differences between the story and no-story conditions with regard to the children’s STEM talk in their immediate reports, *F*(1, 61) = 0.24, *p* = 0.626, *η^2^* = 0.00. Additional correlational analyses revealed that neither parents’ nor children’s story or STEM talk during tinkering was related to children’s STEM- or story-talk during the children’s reflections, *r*s ≤ −0.22, *p*s ≥ 0.083.

### Reminiscing conversation

Recall that all families were invited to reminisce about their tinkering experiences during a second Zoom session. Of the 62 families that participated in the tinkering activity, 45 (73%) engaged in the reminiscing session (21 story-based tinkering, 24 no-story). Five additional families engaged in reminiscing but after a substantially longer delay than the other families (range 150–190 days) and were excluded from the analyses. We found only two differences between families who did (*N* = 45) and did not (*N* = 12) participate in the reminiscing session. As shown in Supplementary Table S2 in the Supplementary material, for parents’ STEM talk during tinkering and children’s story talk in the immediate reflections, the frequencies of talk were higher for those who did not complete the reminiscing conversations compared to those who did.

We hypothesized that the effects of story-based tinkering would be observed in children’s talk about STEM weeks afterward. Specifically, we expected that compared to those in the no-story during tinkering condition, children in the story-based tinkering condition would report more STEM information during parent–child reminiscing weeks after tinkering. On average, the reminiscing conversations occurred 16.33 days after tinkering (range 10–30 days), and we controlled for the delay between tinkering and reminiscing in these analyses. We found that children in the story-based tinkering condition talked significantly more about STEM when reminiscing than those in the no-story condition, *F*(1, 44) = 23.65, *p* < 0.001. *η^2^* = 0.39. For parents’ STEM talk we found no differences by condition, *F*(1, 44) = 2.95, *p* = 0.093, *η^2^* = 0.07.

Finally, we conducted a series of exploratory analyses focusing on the linkages between children’s and parents’ talk during tinkering and children’s recall of the tinkering experience. There were no significant associations between parents’ and children’s STEM talk while tinkering and parents’ and children’s STEM talk when reminiscing, *r*s ≤ 0.23, *p*s ≥ 0.12. Children’s STEM talk in the reflections immediately after tinkering also did not correlate with their STEM talk when reminiscing, *r* = −0.14, *p* = 0.357. Children’s story talk in the reflections immediately after tinkering was significantly related to children’s STEM talk during reminiscing, *r* = 0.34, *p* = 0.024. Additionally, whereas parents’ story talk during tinkering was also not related to children’s STEM talk during reminiscing, *r* = 0.07, *p* = 0.663, children’s talk about the story components during tinkering was significantly related to children’s STEM talk during reminiscing, *r* = 0.46, *p* = 0.002. Therefore, the more story components children mentioned during tinkering, the more children talked about STEM during reminiscing.

## Discussion

### Summary of findings

Taken together, the results of this study provide support for the idea that connecting storytelling and tinkering activities can advance early STEM learning opportunities for children. Prompting families to tell a story during a tinkering activity at home influenced children’s provision of story elements both during tinkering and in their immediate reports of the experience. Therefore, the simple instructions provided before tinkering inviting families to think up a story resulted in differences in storytelling during tinkering. What is more, children in the story-based tinkering group talked the most about STEM both during tinkering, and when reminiscing with their parents about their tinkering experience weeks later. The results add to a growing literature suggesting ways parents and educators can promote children’s STEM talk during STEM-related experiences that improve children’s subsequent retrieval and reporting of STEM information (e.g., Marcus et al., 2017; Pagano et al., 2020; Acosta et al., 2021; Marcus et al., 2021; Sobel et al., 2022).

### Storytelling and informal STEM education

Our focus on oral storytelling during tinkering reflects an effort to consider the role stories can have in supporting academic skills beyond language and literacy (Haden et al., 2023). Although less often the focus of research, oral storytelling can be a crucial way that children gain knowledge at home. Families in both the story and no-story condition included some story elements in their talk during tinkering, which is not surprising when one considers that the video invitation all families viewed included elements of and encouragement to tell a story. Nonetheless, the instructions from the researcher encouraging families in the story-based tinkering condition to think up their story prior to tinkering were additionally effective. Children who heard the story-based instructions included more story elements in their talk during tinkering than those who did not. Moreover, children in the story-based tinkering condition also engaged in more STEM talk than those in the no-story condition. Essentially, by marrying their stories to the reason to tinker (to make a playground ride for a toy friend) children talked more about story and STEM. In this way, our work connects with other recent work suggesting that when individuals are encouraged to tell stories during STEM activities more STEM talk can result (Tzou et al., 2019; Letourneau et al., 2022).

Somewhat unexpectedly, parents did not engage in more story or STEM talk during the activity as a function of story condition. We speculate that regarding story, the brief period when parents and children in the story-based tinkering condition thought up their story might have provided sufficient scaffolding for the children to author their own tale during tinkering. Regarding STEM, parents in both groups engaged in substantial STEM talk, nearly twice that of children. The video that families viewed introducing the activity was aimed at engaging all families in processes of planning, making, testing, and fixing, among other STEM-related practices (e.g., predicting, explaining, comparing), which would have provided a basis for STEM talk across both groups. We saw parents in both groups including elements of STEM talk to support their children’s tinkering, suggesting that the design of the video itself in highlighting the engineering design process encouraged family STEM talk during the home tinkering activity. In fact, this result is consistent with other work in museum settings, suggesting that introducing key STEM principles ahead of engagement in a STEM activity is linked to STEM talk during activities (e.g., Benjamin et al., 2010; Haden et al., 2014; Marcus et al., 2021). That parents in the two groups did not engage differently as a function of our experimental manipulation is in line with past work. There is evidence that when parents are explicitly instructed to use elaborative conversational techniques, for example, they do use them more frequently than uninstructed parents (e.g., Boland et al., 2003; Jant et al., 2014; Chandler-Campbell et al., 2020). But other work further shows that when specific conversational strategies are not called out, there are no differences in parents’ talk as a function of the intervention (Benjamin et al., 2010; Marcus et al., 2017).

The connection between story talk and STEM talk during tinkering is illustrated in the following excerpts of conversations between parents and children in the story-based tinkering condition. In the first, they are building a hot air balloon. The child’s character seems to be at the forefront of the design process, as they are the one making suggestions and modifications to the hot air balloon being built. In particular, the child’s focus on their character is evident when they suggest adding a handle to the balloon, a re-design aimed at meeting a character’s need.

Mother: We need these materials to make it stronger. Okay, now
we are attaching it to this part right [attaches balloon].
Child: Do we want it like that? Or maybe we can move it down. [moves balloon down].
Mother: Okay, so you feel this way. Now here let’s add tape.
Child: They [the character] need something like this [points to door below balloon]. I think that we should add a handle for it to come in.

Likewise, in the next example from another family in the story-based tinkering condition, the child’s suggestion to separate the hot tub from the drying off space by using bricks, and modifying the design to be larger, comes as part of an effort to consider the character’s experience and comfort:

Child: For sure we need something to separate the hot tub.
Mother: Separate the hot tub from?
Child: The place where you dry off. Now I need bricks of …like these to make it big. Good?
Mother: Good!

Relative to the no-story group, children in the story-based tinkering group also talked more about story, but not STEM, in their immediate reports. Talking about the story in connection with tinkering in these reports may have served a consolidation function, further cementing the link between the story and STEM. The following example of a child in the story-based tinkering group illustrates how the story characters motivated making and iteration of their design:

Researcher: So, can you tell me about how you tested all these different projects to make sure they worked?

Child: Sure. The swing was first try. And the problem for the see-saw is that the people, the ponies fell out and then the seats came off. So we used packaging tape for this one and we used um rubber bands for both of them. And the rubber bands are bonus to a seat belt. We got it on the second try. The problem with this was that Mary couldn’t stand up so we had to keep this in the middle and works as a ladder.

The immediate reports revealed that children in both the story and no-story groups were able to report similar amounts of STEM information in the immediate reflections. The children were recalling quite a bit of STEM content about their tinkering experiences. It is likely that the researcher’s questions eliciting the children’s reflections provided support for reporting STEM-related information, benefiting both groups of children to the same extent immediately after tinkering.

When we considered the results of reminiscing conversations that occurred several weeks after tinkering, we did find the anticipated differences in children’s reports of STEM information. Compared to those in the no-story group, children in the story-based tinkering group talked more about STEM when reminiscing with their parents weeks after their tinkering experience. Parents in the two groups did not differ in the story or STEM talk during reminiscing, pointing to the unique role that the story-based tinkering had on children’s abilities to retrieve and report their experiences later. Other work has similarly found delayed effects of enriched informal tinkering and building experiences, such that differences in recall of STEM-related information are most evident weeks later (e.g., Benjamin et al., 2010; Marcus et al., 2021). Likewise, in this study, the connection between the story and the STEM information forged during the tinkering activity and immediately afterward benefited children’s later recall of STEM. The following excerpt from a family in the story-based tinkering condition illustrates how the connection between story and STEM during tinkering was further manifest in their subsequent remembering of the experience:

Child: We used the cups.
Mother: Oh yeah! We cut the cups.
Child: We kinda…
Mother: Because it think it was three cups, wasn’t it?
Child: And then the other part that was taped came off.
Mother: Oh yeah.
Child: Then we used it as a door.
Mother: Oh yeah, that’s right.
Child: I think that was really cool.
Mother: And how did they get to the slide? How did they get up there?
Child: They used the steps (laughs) that you built. They have to have like enormous feet or have really long legs.
Mother: So that didn’t work that well did it.
Child: They had to jump up the stairs. Bounce down.
Mother: …And the slide was good, right? It worked.
Child: Mmhmm.
Mother: And they were contained at the bottom, right?
Child: Yeah. And then they could open the door. Otherwise, they’d be just trapped in there. A pile of unicorns.

## Limitations, implications, and future directions

It is important to consider a range of ways that children engage with stories when thinking about how stories can support informal STEM learning. Stories are infused into many of the kinds of activities that children engage in at home, from videogames and television viewing, to book reading. But the scant attention to oral storytelling may limit insights into the ways that stories as everyday practices can provide mechanisms for children’s STEM learning, especially for children from cultural backgrounds with rich oral traditions (Haden et al., 2023). One contribution of our work to educational practice is in showing how oral storytelling can increase STEM learning opportunities based in hands-on activities at home. Unfortunately, a limitation of our online work during the COVID-19 pandemic is that our recruitment strategy did not yield a diverse sample in terms of cultural background, socioeconomic status, or parental education. We have addressed this limitation, in part, in other work that focuses on Latine families’ oral storytelling and tinkering at home (e.g., Acosta, 2022). Gaining understanding of how families from different cultural communities might benefit from online museum programming is important when we think about its potential to increase access and opportunities for STEM learning for all families, including those who, for various reasons, are not regular museum visitors.

It is encouraging that the video invitation to tinker at home was itself effective in engaging families in storytelling and STEM talk. We know from prior work that the mix of parent–child STEM-related talk observed during tinkering depends on the design of the tinkering activity (Pagano et al., 2020). The video invitation in this study emphasized the engineering design process, as well as mathematics (measuring) and science (explanations), and this emphasis was reflected in the conversations we observed. With the transition back to the museum, we have been considering with our museum partners how what was learned from the digital program can inform museum practice. Families do not usually view a video before engaging in tinkering in an exhibit space. Likewise, the facilitation provided by museum educators in tinkering exhibits is often briefer in introducing the activity, and sometimes turns intermittent as families progress through the activity. Families in exhibits are faced with a mix of familiar and potentially unfamiliar materials. There are also other families and models throughout the space that families might use to gain further information about what there is to do and learn. Due to these differences and others, it is crucial to take steps to study whether the current findings translate across settings, specifically from home to the museum. With the Chicago Children’s Museum having reopened, we are engaging in this work now. From the current study, a further implication for practice is that connecting stories and hands-on activities from the outset of the activity can increase the opportunities for STEM learning these activities provide.

Additionally, our work encourages practices that invite reflection on STEM learning to advance and reveal learning. Moreover, the current findings regarding children’s retention of STEM information support further consideration of whether STEM learning promoted by oral storytelling *transfers* across settings—for example, from home to museum, and museum to home. Past work does suggest that STEM learning at home is related to STEM learning in school settings (e.g., Skwarchuk et al., 2014; Junge et al., 2021; Westerberg et al., 2022). Less is known about how learning in museums transfers beyond museum walls, but the work that does exist (e.g., Marcus et al., 2017, 2021) recommends this is a promising avenue for future work. The findings from this study indicate that storytelling during STEM activities may be especially important in promoting such transfer, as evidenced by the fact that pairing storytelling and tinkering led to more durable and retrievable memories for the STEM learning experiences.

## Data availability statement

The raw data supporting the conclusions of this article will be made available by the authors, without undue reservation.

## Ethics statement

The studies involving human participants were reviewed and approved by Loyola University Chicago. Written informed consent to participate in this study was provided by the participants’ legal guardian/next of kin.

## Author contributions

CH secured funding and with GS conceptualized the idea for this study. GS was responsible for the data collection and conducted the coding with SS. MM conducted the statistical analyses. CH, MM, and GS contributed to the writing. All authors read, edited, and approved the submitted version of the manuscript.

## Funding

This research was supported by the National Science Foundation (NSF) under a collaborative grant DRL#1906940 to CH (PI Loyola University Chicago, including a subaward to MM, Roosevelt University), #1906808 to David Uttal (PI Northwestern University), and #1906839 Tsivia Cohen (PI Chicago Children’s Museum).

## Conflict of interest

The authors declare that the research was conducted in the absence of any commercial or financial relationships that could be construed as a potential conflict of interest.

## Publisher’s note

All claims expressed in this article are solely those of the authors and do not necessarily represent those of their affiliated organizations, or those of the publisher, the editors and the reviewers. Any product that may be evaluated in this article, or claim that may be made by its manufacturer, is not guaranteed or endorsed by the publisher.

## Footnotes

^1^ www.eie.org

^2^ https://www.youtube.com/watch?v=PRTwI9vDFoM

^3^ onetonline.org
