# Supplementary material for: Promoting children’s science, technology, engineering, and mathematics learning at home through tinkering and storytelling
Source: Front Psychol. 2023 May 3;14:1146063. doi: 10.3389/fpsyg.2023.1146063 (PMC10189131; doi:10.3389/fpsyg.2023.1146063)
Supplement: Supplementary file 1 [file Table_1.docx]

**Supplementary Materials for “Promoting Children’s STEM Learning at Home Through Tinkering and Storytelling”**

Table S1

*Comparison Between Families in the Story-Based and No Story Tinkering Conditions:*

|  | Story-Based  (*n* = 29) | |  | No Story  (*n* = 33) | |  | Condition | | |
| --- | --- | --- | --- | --- | --- | --- | --- | --- | --- |
|  | *M* | *SD* |  | *M* | *SD* |  | *F* | *p* | *η^2^* |
| Children’s Prior Experiences | |  |  |  |  |  |  |  |  |
| Building and making | 4.75 | (1.84) |  | 4.45 | (1.54) |  | .47 | .498 | .01 |
| STEM kits and toys | 5.29 | (1.74) |  | 4.58 | (1.97) |  | 2.19 | .144 | .04 |
| Arts and crafts | 5.54 | (1.40) |  | 5.21 | (1.36) |  | .83 | .365 | .01 |
| Housework repairs | 4.57 | (2.13) |  | 4.39 | (1.84) |  | .12 | .728 | .00 |
| Spatial activities | 5.39 | (1.97) |  | 4.48 | (2.02) |  | 3.14 | .082 | .05 |
| Read books | 6.75 | (.84) |  | 6.58 | (1.06) |  | .49 | .486 | .01 |
| Write stories | 5.22 | (1.73) |  | 4.88 | (1.87) |  | .42 | .519 | .01 |
| Play/shows | 3.30 | (1.80) |  | 3.71 | (2.10) |  | .50 | .482 | .01 |
| Pretend play | 5.26 | (1.89) |  | 4.96 | (1.92) |  | .30 | .589 | .01 |
| Talk of past activities | 6.70 | (.77) |  | 6.38 | (1.21) |  | 1.17 | .285 | .03 |

*Note.* Children’s prior experiences were reported by parents on a 1–7 scale.
