# Supplementary material for: Promoting children’s science, technology, engineering, and mathematics learning at home through tinkering and storytelling
Source: Front Psychol. 2023 May 3;14:1146063. doi: 10.3389/fpsyg.2023.1146063 (PMC10189131; doi:10.3389/fpsyg.2023.1146063)
Supplement: Supplementary file 2 [file Table_2.docx]

Table S2

*Comparison Between Families Who Did and Did Not Complete the Reminiscing Conversations*

|  |  | Reminiscing Conversations | | | | |  |  | | |
| --- | --- | --- | --- | --- | --- | --- | --- | --- | --- | --- |
|  |  | Completed  (*n* = 45) | |  | Did Not Complete  (*n* = 12) | |  |  | | |
|  |  | *M* | *SD* |  | *M* | *SD* |  | *F* | *p* | *η^2^* |
| Demographics | |  |  |  |  |  |  |  |  |  |
|  | Child age | 8.00 | 1.67 |  | 7.75 | 2.14 |  | 0.19 | .665 | .00 |
| Story talk during tinkering | |  |  |  |  |  |  |  |  |  |
|  | Parents’ story talk | 5.13 | 2.51 |  | 5.75 | 3.47 |  | 0.48 | .490 | .01 |
|  | Children’s story talk | 6.16 | 4.17 |  | 6.00 | 3.98 |  | 0.01 | .908 | .00 |
| STEM talk during tinkering | |  |  |  |  |  |  |  |  |  |
|  | Parents' STEM talk | 12.80 | 3.14 |  | 15.08 | 3.15 |  | 4.99 | .030 | .08 |
|  | Children's STEM talk | 6.27 | 2.15 |  | 6.67 | 2.57 |  | 0.30 | .584 | .01 |
| Children’s talk immediately after tinkering | |  |  |  |  |  |  |  |  |  |
|  | Children's story talk | 9.44 | 2.64 |  | 11.33 | 2.46 |  | 4.97 | .030 | .08 |
|  | Children’s STEM talk | 10.42 | 2.91 |  | 10.33 | 4.05 |  | 0.01 | .932 | .00 |
